# Meckel’s Diverticulum − a Congenital Defect of the Gastrointestinal Tract Underestimated in Differential Diagnostics. Own Experience

**DOI:** 10.34763/devperiodmed.20172101.3842

**Published:** 2017-05-29

**Authors:** Jolanta Pietrzak, Anna Obuchowicz, Dariusz Majda, Andrzej Kiedos

**Affiliations:** 1 Department of Paediatrics in Bytom, School of Health Sciences in Katowice, Medical University of Silesia, Katowice, Poland; 2 Department of Paediatric Surgery, Specialist Hospital No 2 in Bytom Poland

**Keywords:** Meckel’s diverticulum, intestinal obstruction, child, uchyłek Meckela, niedrożność jelit, dziecko

## Abstract

Meckel’s diverticulum is a vestigial remnant of the omphalomesenteric duct. It is the most frequent defect of the gastrointestinal tract and is present in approx. 2% of the population: more often in boys than in girls, at a 3:1 ratio. Meckel’s diverticulum causes clinical symptoms only in 4-6% of individuals. It is situated approximately 60-100 cm away from the Bauhin’s valve. The wall of Meckel’s diverticulum consists of all layers typical of the small intestine. It is usually approx. 2-3 cm long, but it can reach lengths of over 10 centimetres. It is usually set on a narrow base. Related symptoms usually occur by the 2^nd^-3^rd^ year of life and have an acute character. They may occur in older children, which must be remembered in differential diagnosis. An example can be the case of an 8.5-year-old boy with symptoms indicating obstruction of the gastrointestinal tract in which a large, atypically accreted and partly twisted Meckel’s diverticulum was found with strangulation of the small intestine between the diverticulum and the mesentery.

## Streszczenie

Uchyłek jelita krętego zwany uchyłkiem Meckela, jest pozostałością przetrwałego − częściowo lub całkowicie − przewodu żółtkowego. Jest najczęstszą wadą przewodu pokarmowego. Występuje u około 2% osób, częściej u chłopców niż u dziewczynek, w stosunku 3:1. Uchyłek Meckela jedynie u 4-6% osób wywołuje objawy kliniczne. Umiejscowiony jest w odległości około 60-100 cm od zastawki krętniczokątniczej Bauhina. Ściana uchyłka składa się ze wszystkich warstw typowych dla jelita cienkiego Jego długość wynosi zwykle około 2-3 cm, ale może osiągać również kilkanaście centymetrów. Osadzony jest zwykle na wąskiej podstawie. Objawy z nim związane występują najczęściej do 2-3 roku życia i mają charakter ostry. Istnieje możliwość ich wystąpienia u starszych dzieci, o czym należy pamiętać w diagnostyce różnicowej. Przykładem może być leczony przez nas 8,5-letni chłopiec z objawami wskazującymi na niedrożność przewodu pokarmowego, u którego w czasie laparotomii stwierdzono duży, nietypowo przyrośnięty i częściowo skręcony uchyłek Meckela, z zadzierzgnięciem jelita cienkiego między uchyłkiem a krezką jelita.

Meckel’s diverticulum is a vestigial remnant of the omphalomesenteric duct (*ductus omphaloentericus*). It is the most frequent defect of the gastrointestinal tract and is present in approximately 2% of the population, more often in boys than in girls, at a 3:1 ratio [1, 2, 3, 4]. It creates a bulge of the small intestine at a distance of approximately, 60-100 cm from the Bauhin’s valve on the antimesentery side of the intestine. It is a real diverticulum. The wall of Meckel’s diverticulum consists of all layers typical of the small intestine [1, 4]. It is usually approx. 2-3 cm long, but it can also reach lengths of over 10 centimetres. It is usually set on a narrow base [2, 5, 6]. In 10% of the patients, the diverticulum is connected with the navel or the mesentery with a strip of connective tissue [1, 5, 6]. This defect is usually asymptomatic [2, 7, 8]. It causes symptoms in only approximately 4-6% of individuals, usually by the 2nd or 3rd year of life [1, 2, 9], and they depend, among other things, on the size of the diverticulum [10, 11]. Larger diverticula are more often sources of complications [6, 10, 11].

In 30-50% of patients, the diverticulum is lined with ectopic tissue (mucosa of the stomach, duodenum, large intestine, biliary tract and pancreatic tissue), which may cause complications in the form of ulcers, bleeding or perforation with pathological symptoms in the abdominal cavity [1, 2, 3].

The most frequent symptoms include ones imitating acute appendicitis, bleeding from the gastrointestinal tract, recurrent acute abdominal pain caused by in$ammation of the ectopic mucosa, symptoms of obstruction of the gastrointestinal tract, symptoms of localized or diffuse peritonitis in the case of (very rare) perforation of the diverticulum [4, 5, 8, 9, 11].

Bleeding and intussusception due to the existence of Meckel’s diverticulum are more frequent in children under 2 years of age, and symptoms of obstruction of the gastrointestinal tract and inflammation of Meckel’s diverticulum occur more often in adults (6, 11, 12). Cases of purulent in$ammatory conditions of the diverticulum in children are described sporadically [10, 13]. Tumours can develop in Meckel’s diverticulum only very rarely, e.g. carcinoid, leiomyosarcoma [2].

Diagnosis is often difficult due to a lack of specific symptoms [10, 12, 14]. Meckel’s diverticulum is usually diagnosed during planned or urgent surgeries for other reasons, or when complications occur that are related to its presence and are an indication for a laparotomy [1, 4, 5, 7, 10]. A lot of authors claim that laparoscopy is useful in such situations due to the low invasiveness of this method and its cosmetic advantages as compared to laparotomy [3, 6, 15, 16, 17]. During diagnostics or bleeding from the gastrointestinal tract, diverticula, which contain ectopic tissue of the stomach or pancreas, can be detected on the basis of a scintigraphy examination using technet ^99m^Tc [2, 6, 18, 19]. Some authors have described cases of diagnosing the diverticulum using a CT or MRI scan [1, 15, 19, 20], capsule enteroscopy [2] and also using double-balloon enteroscopy [2, 21]. An abdominal ultrasound is not useful in these cases [10].

In differential diagnosis we should be aware of the non-specific symptoms in Meckel’s diverticulum. One should also take into consideration the possibility of Meckel’s diverticulum that is anatomically atypical, and therefore may be more difficult to diagnose. An example of such a situation may be the case of an 8.5-year-old boy with symptoms indicating obstruction of the gastrointestinal tract, in which a large, atypically accreted and partly twisted Meckel’s diverticulum was found with strangulation of the small intestine between the diverticulum and the mesentery.

In the boy described above, vomiting occurred repeatedly for 2 days, and it first included contents of the digestive system. Later the vomited substance became watery and bile-coloured. He received an anti-emetic drug, which did not have any effect. From the second day, severe spasmodic abdominal cramps occurred that were characterized as becoming more intense in the lying position and receded in the sitting position with the upper body bent forward. The boy did not have fever and had normal stools twice.

**Fig. 1:**
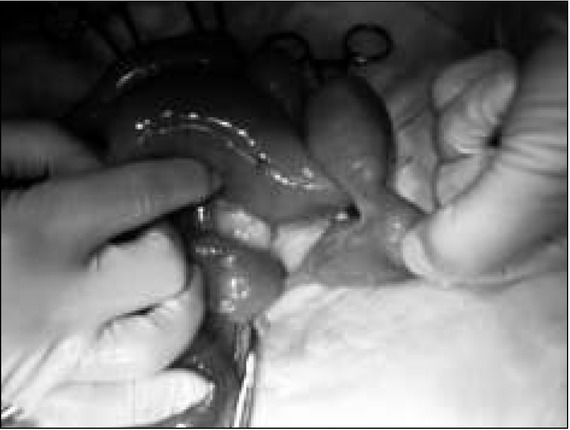
Distended small bowel loop and Meckel's diverticulum accreted to the back wall of the abdomen. Ryc. 1. Rozdęte pętle jelita i uchyłek Meckela z pasmem przyrośniętym do tylnej ściany jamy brzusznej.

**Fig. 2:**
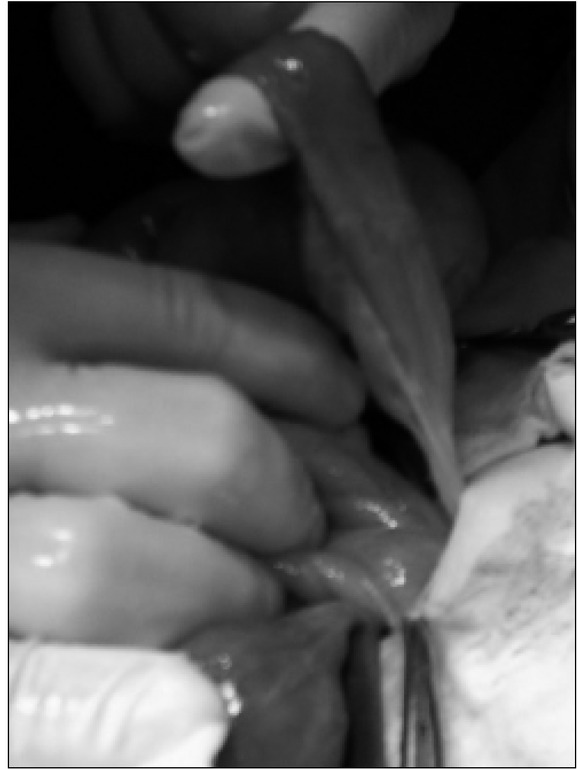
Meckel’s diverticulum during preparation. Ryc. 2. Uchyłek Meckela w trakcie preparowania.

**Fig. 3:**
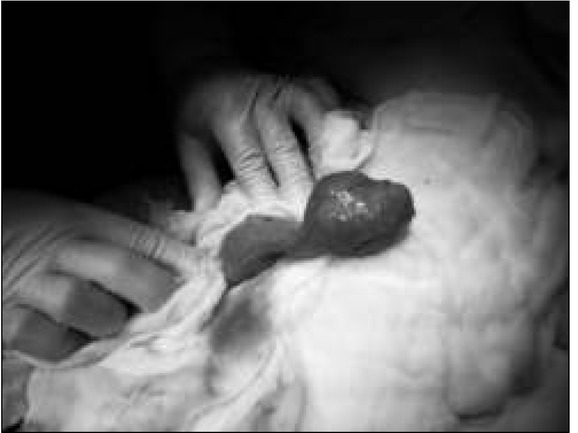
Meckel’s diverticulum dissected. Ryc. 3. Wypreparowany uchyłek Meckela.

**Fig. 4:**
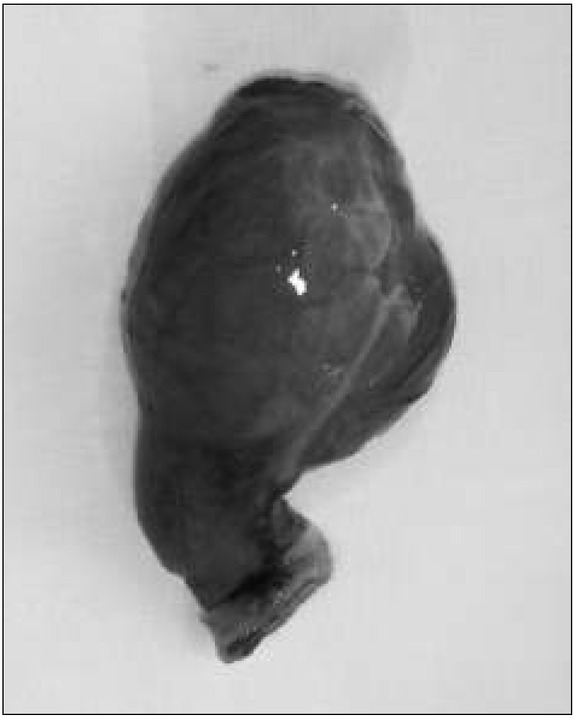
Removed Meckel’s diverticulum measuring 6x4 cm. Ryc. 4. Usunięty uchyłek Meckela o wymiarach 6x4 cm.

There was a small amount of free $uid in the small pelvis, bloated intestinal loops, single enlarged lymph nodes, scant peristalsis in the ultrasonography result. The boy was transferred to the Children’s Surgical Ward with a suspected obstruction of the gastrointestinal tract and a laparatomy was performed urgently. A normal appendix was found, which was typically removed. Revision of the abdominal cavity revealed significant bloating of the jejunum. The bloating was caused by a large, sack-like Meckel’s diverticulum (dimensions: 6x4 cm), partially twisted, accreted to the back wall of the abdominal cavity, with strangulation of the small intestine loop between the Meckel’s diverticulum and the mesentery [Fig. 1, 2, 3, 4].

In the boy described, unrestrained vomiting, which lasted for over 24 hours without any other clinical symptoms caused difficulties in making a proper diagnosis. Vomiting without pain symptoms is rare and indicates the presence of Meckel’s diverticulum. There are few such reports in the literature [1, 15]. The publication by Codrich et al. deserves special attention, as it describes the case of a 7-year-old boy with recurring vomiting (for a year) and cramping abdominal pain in the naval area caused by the presence of Meckel’s diverticulum [1]. Vomiting preceding abdominal pain, which was the first symptom of the obstruction caused by Meckel’s diverticulum, was described by Itriyeva et al. [14]. Similar symptoms – vomiting that was preceded by colic pain in the umbilical region lasting for more than a day, was experienced by the boy we are describing. However, those symptoms progressed and obstruction of the gastrointestinal tract was soon diagnosed. The relationship between the position of the patient’s body and pain symptoms deserves special attention, as it was most likely connected with anatomical conditions and the fact that the diverticulum was accreted to the back abdominal wall and partially twisted.

The discovery of Meckel’s diverticulum described in this study deserves attention due to the rare coexistence of its atypical features: the size and shape, accretion to the back abdominal wall and the occurrence of partial twisting. One should also notice that, despite the twisting and strangulation of the small intestine loop between the diverticulum and the mesentery, no ischemia or gangrene of Meckel’s diverticulum or the small intestine occurred, despite the fact that surgical treatment was used only during the second day in which the symptoms persisted.

If Meckel’s diverticulum is present, the intestinal obstruction is usually related to its in$ammation or existence of a connective tissue strip connecting the diverticulum with the navel, which is a factor leading to the twisting of the intestine with strangulation [3, 5, 6, 8]. None of these causes were found in the patient, and the obstruction was related to its anatomy − the diverticulum was large and atypically accreted to the back wall of the abdominal cavity, which caused strangulation of the small intestine. Moreover, due to its large dimension, the diverticulum became partly twisted. Reports on this complication are rarely found in the literature [3, 11, 15, 22]. Nose et al., on the basis of a literature overview (performed in 2013), reported only 6 such cases in children and 20 in adults [3]. Axial twisting may contribute to necrosis and perforation, and this mostly applies to long diverticula with a narrow base [3, 15, 22], i.e. with a di&erent structure than that described in the boy. It can be suspected that severe vomiting led to the partial twisting of the diverticulum.

In some cases, the symptoms imply appendicitis [3, 6, 12, 16, 22]. Severe pain occurs in the right lower abdomen and sporadic vomiting is observed [3, 6]. Laboratory tests and imaging scans (RTG, CT, ultrasound scans of the abdominal cavity) can also imply acute appendicitis [3, 6]. In the case described herein, vomiting was the leading symptom, followed by severe abdominal pain which, however, was located in the lumbar abdominal region and was reduced by leaning the body forward.

As in other cases of this kind that were described in the literature, both Meckel’s diverticulum and the morphologically and histopathologically unchanged appendix were removed [6, 15]. It is suggested that all patients with appendicitis should be examined for the presence of Meckel’s diverticulum. If it is found, it should also be removed [4, 6, 15, 23, 24]. This suggestion is confirmed by statistical data, according to which, complications occur in 12% of cases and the mortality is 2% if a pathological diverticulum is removed; these numbers decrease for the removal of an asymptomatic diverticulum that are discovered by accident: the frequency decreases to 2% and mortality to 1% [6, 23]. Some authors imply that the presence of Meckel’s diverticulum should additionally be taken into consideration in children with recurrent intussusception [14].

Despite the fact that gastric mucosa was found in the histological examination of the removed diverticulum described, this fact was not the cause of the complications.

In 5-10% of patients with Meckel’s diverticulum, other defects of the umbilical cord co-exist [5, 25]. In the patient presented, only delayed navel healing occurred during the neonatal period. On the basis of available literature, it is difficult to decide whether this fact can be related to the existence of Meckel’s diverticulum. After the overview of 26 patients with Meckel’s diverticulum, Chen et al. inform that irregularities in the navel region (hernia, patent omphalomesenteric duct) occurred in 23% of the patients [25].

## Conclusion

Meckel’s diverticulum is rarely recognizable, despite being the most frequent congenital defect of the gastrointestinal system. The paediatrician should take into account the possibility of its presence and related complications in each patient with pathological symptoms in the abdominal cavity, and the surgeon should realize that it can have atypical anatomical features and location.
